# In Vitro Metabolism of Phenylspirodrimanes Derived from the Indoor Fungus *Stachybotrys*

**DOI:** 10.3390/toxins14060395

**Published:** 2022-06-08

**Authors:** Viktoria Lindemann, Annika Jagels, Matthias Behrens, Florian Hübner, Hans-Ulrich Humpf

**Affiliations:** Institute of Food Chemistry, Westfälische Wilhelms-Universität Münster, 48149 Münster, Germany; viktoria.lindemann@uni-muenster.de (V.L.); annika.jagels@whitney.ufl.edu (A.J.); mattbehrens@uni-muenster.de (M.B.); florian.huebner@uni-muenster.de (F.H.)

**Keywords:** *Stachybotrys*, phenylspirodrimanes, mycotoxins, indoor, metabolism, high-resolution mass spectrometry

## Abstract

Fungi belonging to the genus *Stachybotrys* are frequently detected in water-damaged indoor environments, and a potential correlation between emerging health problems of inhabitants of affected housing and the fungi is controversially discussed. Secondary metabolites (i.e., mycotoxins) produced by *Stachybotrys*, such as the highly toxic macrocyclic trichothecenes (MCTs), are of potential concern to human health. The present study, however, focused on the potential effects of the more broadly and abundantly formed group of phenylspirodrimanes (PSDs). The phase I and II metabolism of four structurally different PSDs were investigated in vitro using hepatic models in combination with high-performance liquid chromatography high-resolution mass spectrometry (HPLC-HRMS) analysis. In addition to metabolite detection by HRMS, isolation and structure elucidation by nuclear magnetic resonance spectroscopy (NMR) was part of the conducted study as well.

## 1. Introduction

Fungi belonging to the genus, *Stachybotrys*, are considered ubiquitously distributed in the environment but are mainly found indoors after water damage [1]. Due to its characteristic morphology, *Stachybotrys* carries the nickname “black mold” [2]. It was first described in 1837 after isolation from a wallpaper in Prague [3] and is primarily known for its involvement in cases of illness of farm workers and animals in Eastern Europe in the first half of the 20th century. Symptoms of stachybotryotoxicosis range from inflammation and abscesses in the gastrointestinal and respiratory tract to liver damage [4,5,6]. In 1986, however, *Stachybotrys* gained attention after the first occurring human case of stachybotryotoxicosis without an agricultural background in Chicago [7]. Further cases occurred in the 1990s in the Cleveland area. Thirty-seven children suffered from toxicosis and pulmonary bleeding after indoor exposure to the fungus. For 12 children, the disease was fatal [7,8]. In all described examples, exposure to the affected people took place through indoor *Stachybotrys* infestation. Modern scientific research, therefore, focuses on a more detailed exposure assessment and on the characterization of the potential health effects [9]. Nevertheless, the actual correlation between exposure to *Stachybotrys* species and (respiratory) disease is still not causally proven and is controversially discussed [10].

**Figure 1:**
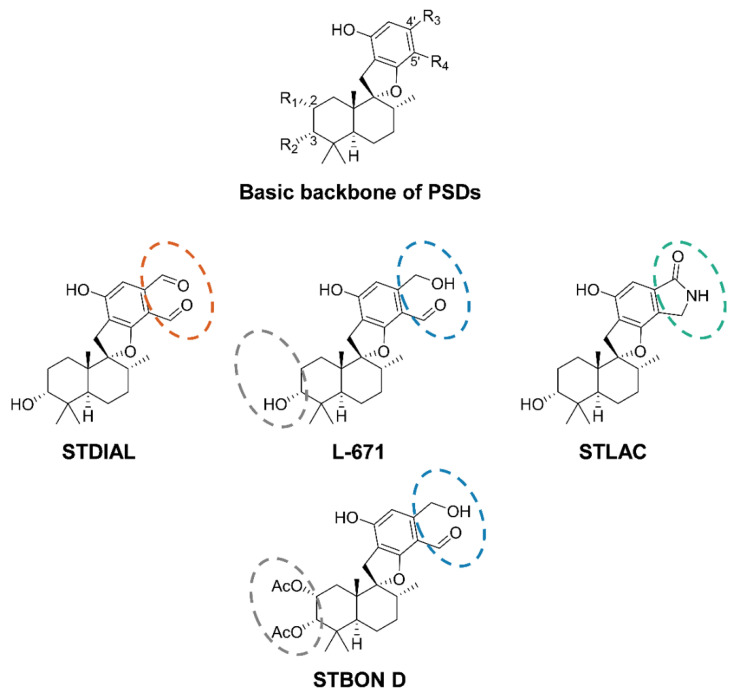
Chemical structures of the basic backbone of phenylspirodrimanes (PSDs) and of the four PSDs stachybotrydial (STDIAL), L-671,667 (L-671), stachybonoid D (STBON D), and stachybotrylactam (STLAC), which were applied to hepatic metabolism studies in the presented project. Structural differences at the basic backbone are highlighted in grey and varying substituents at positions 4′ and 5′ by colored framings.

It is suspected that stachybotryotoxicosis is not solely caused through contact to spores and fungal fragments but also by secondary metabolites such as mycotoxins [6]. Secondary metabolites are compounds with low molecular masses, which, in contrast to primary metabolites, are not directly required for the growth of the producing organism. Fungi in particular are able to form a multitude of secondary metabolites [11]. The previously mentioned mycotoxins are a diverse group of compounds that can cause inflammation and nephrotoxic, hepatotoxic, and genotoxic effects in mammals [12]. Especially, the highly toxic macrocyclic trichothecenes (MCTs), which represent one of three main compound classes produced by *Stachybotrys*, are a suspected cause of potential diseases [13,14]. Among the MCTs, satratoxins are often produced by *Stachybotrys* at the highest concentrations [15,16]. MCTs are strong inhibitors of protein biosynthesis [17] and show cytotoxic and immunosuppressive effects. The half maximal inhibitory concentrations (IC_50_) for satratoxins are in the nanomolar range [18]. However, MCTs are only produced by a certain chemotype of *Stachybotrys*, which accounts for about one-third of detected *Stachybotrys* indoor infestations [1]. The same applies to the second group of *Stachybotrys* toxins: atranones are even less frequently detected and usually produced in low concentrations [1]. Nevertheless, it was proven that atranones can cause inflammatory effects [19,20]. More relevance concerning the distribution and produced levels can be attributed to phenylspirodrimanes (PSDs), which are the third group of the main secondary metabolites. They are produced by all known *Stachybotrys* species [21,22]. As hybrid synthesis products, PSDs are synthesized by polyketide synthases as well as by the terpene biosynthetic pathway and show a high structural diversity [23]. The chemical structure of the PSD backbone, as well as of the four PSDs, which were investigated in this study, are shown in Figure 1.

Depending on the substituents in positions R_3_ and R_4_ of the basic backbone, the formation of a ring structure is possible and, for example, the conversion to lactones can be observed in aqueous solutions for structures bearing two aldehyde groups at positions 4′ and 5′ [24,25]. Especially, PSDs with dialdehyde moieties are known to have immunosuppressive effects [26]. For some derivatives, cytotoxicity data are available and with IC_50_ values in the two-digit micromolar range, the tested PSDs are less cytotoxic compared to MCTs [27,28]. Based on these results, PSDs do not seem to be the sole cause for stachybotryotoxicosis. However, since MCTs are only present in low concentrations and not formed by all species, combinatorial effects of the mycotoxin groups have to be taken into consideration [21]. Furthermore, the influence of metabolism on the toxicity of the mentioned mycotoxins and, therefore, on stachybotryotoxicosis has been studied to a minor degree. It could, however, be of relevance for human exposure, as metabolism can involve both a detoxification as well as a toxification [29]. The data available on the metabolism of all three substance groups are limited. For satratoxin G, a fast biotransformation is postulated [30]. The metabolism of atranones and PSDs in vitro and in vivo have not been investigated yet. Overall, it is therefore difficult to assess to which extent the various compound classes are involved in or responsible for the clinical symptoms of stachybotryotoxicosis. Especially, the role of PSDs needs further exploration, since in addition to metabolism, the cytotoxicity of different representatives of this structural class is not yet sufficiently described.

The purpose of the presented studies was therefore to gain insight into the metabolism of PSDs. With this objective, four structurally diverse PSDs were applied to in vitro hepatic phase I and II (pI and pII) metabolism models. Identification and characterization of possible metabolites were carried out by high-performance liquid chromatography coupled to high-resolution Orbitrap mass spectrometry (HPLC-Orbitrap-HRMS) or quadrupole time-of-flight (QTOF) HRMS and nuclear magnetic resonance spectroscopy (NMR).

## 2. Results and Discussion

### 2.1. Metabolism Studies

In order to study the in vitro phase I and II (pI and pII) metabolism of PSDs, hepatic microsomes and cytosol of humans were used due to the possible exposure to PSDs in residential settings. Additionally, horse microsomes and cytosol were deployed to examine the possible differences in metabolism and as this species has historically shown an increased sensitivity towards the genus *Stachybotrys* [6]. The studies were carried out exemplarily with stachybotrydial (STDIAL), L-671,667 (L-671), and stachybotrylactam (STLAC), which share the PSD backbone but show structural differences at the phenolic moiety of the molecules (see the orange, blue, and green framings in Figure 1). Furthermore, stachybonoid D (STBON D) was included in the studies because of its varying substituents on the drimane structure (compare the grey framing in Figure 1). Details on the performed metabolism experiments are presented in Tables S1–S3 in the Supplementary Materials.

#### 2.1.1. Phase I Metabolism of PSDs in Human and Horse Liver Microsomes

**Figure 2:**
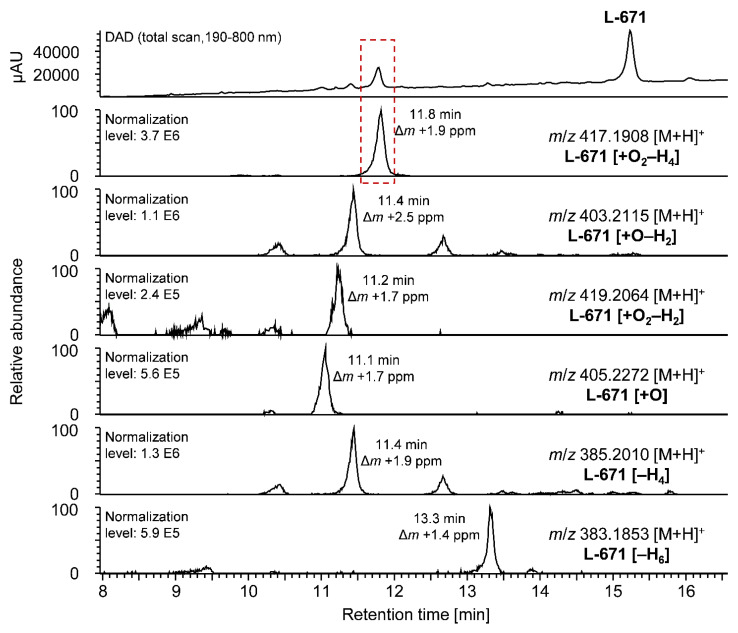
HPLC-DAD-Orbitrap-HRMS chromatograms of the hepatic pI metabolism of L-671 in human liver microsomes and extracted ion chromatograms (XICs) (acquired with a mass tolerance of 5 ppm) of potential metabolites of L-671. In the case of multiple occurring signals, the mass deviation (Δ*m*) of the most abundant signal is given.

For studies on the hepatic pI metabolism, PSDs were incubated with liver microsomes as described in Section 4. Sample analysis was performed by HPLC-diode array detection (DAD)-Orbitrap-HRMS. For identification of metabolites, Orbitrap-HRMS data were processed and annotated using a self-created mass list. The underlying considered (combinations of) metabolic reactions and the correlating changes in sum formula of the investigated PSDs as well as the applied nomenclature of possible metabolites can be found in the Supplementary Materials in Tables S4 and S5. Metabolites were identified by comparing the calculated exact masses, mainly of the [M+H]^+^ adducts, with the recorded data. In the case of STBON D, the [M-H_2_O+H]^+^ adduct was also taken into consideration, since an in-source loss of water is common for this analyte. Exemplarily, the results of the metabolism of L-671 by human microsomes are presented in Figure 2. The DAD and the extracted ion chromatograms (XICs) of L-671 and its identified metabolites are shown in Figure 2. The nomenclature of the metabolites was based on the difference in the sum formula compared to the parental compound (given in square brackets without charge). Three metabolites (i.e., L-671 [+O_2_–H_4_], L-671 [+O–H_2_], and L-671 [+O_2_–H_2_]) were formed by the addition of oxygen in combination with a desaturation. L-671 [+O] emerged when only oxygen was added to the sum formula of the parental compound, whereas the other two metabolites (i.e., L-671 [–H_4_] and L-671 [–H_6_]) were created by desaturation.

All identified metabolites showed a higher hydrophily in comparison to the respective parental compound, as they eluted earlier on the used reversed-phase chromatography system (Figure 2). In the DAD chromatogram, in addition to the signal of the parental compound, L-671, one other intensive signal was noticeable at 11.8 min (red rectangle) representing the metabolite L-671 [+O_2_–H_4_], the result of the two-fold addition of oxygen to L-671 in combination with desaturation. All other metabolites showed only small intensities in the DAD chromatogram, indicating that L-671 [+O_2_–H_4_] was the main metabolite formed in the pI experiments. This was confirmed in the HPLC-Orbitrap-HRMS-chromatograms, as the XIC of L-671 [+O_2_–H_4_] showed the highest intensity compared to the other metabolites. The other potential metabolites of L-671 did not show eminent signals in the DAD chromatogram but can be identified in the corresponding XICs with high mass accuracy. Like L-671 [+O_2_–H_4_], the metabolites L-671 [+O–H_2_] and L-671 [+O_2_–H_2_] were formed by the addition of oxygen in combination with desaturation. For the sum formulas of these metabolites, it can be noticed that the XICs of the exact masses reveal more than one peak. This indicates the formation of the metabolic reaction at different reaction sites of L-671 or the formation of isomers. The metabolite L-671 [+O] at 11.1 min was formed by an addition of oxygen to L-671, whereas the metabolites L-671 [–H_4_] and L-671 [–H_6_] were formed by desaturation of the parental compound. In the case of L-671 [–H_4_], again, several signals can be recognized. The main peak eluted at 11.4 min. Concerning this potential metabolite, it must be noted that despite a desaturation process of L-671, it could also be the metabolite L-671 [+O–H_2_], which underwent an in-source loss of water during ionization. The XIC of L-671 [–H_6_], however, showed only one signal with the *m*/*z* 385.2010 at a retention time of 13.3 min.

Concerning the conversion of the other investigated PSDs in the human hepatic pI metabolism model, the formation of comparable metabolites was observed. However, it was noticeable that a sole desaturation (PSD [–H_4/6_]) was only detected for L-671. The other potential metabolites were found for all PSDs with varying signal intensities. STDIAL [+O–H_4_] was identified as the main pI metabolite of STDIAL, whereas higher signal intensities were detected for the oxidized metabolites ([+O]) for STLAC and STBON D. Though, it should also be noted that the *m*/*z* 487.2326 of STBON D [+O] could also refer to the [M+H]^+^ adduct of a desaturated metabolite, since both metabolites share the same *m*/*z*. In this case, further studies are needed for a precise structural assignment. For a detailed overview of the resulting metabolites and their experimental data, please refer to Table S6 and Figures S1–S3 in the Supplementary Materials.

**Figure 3:**
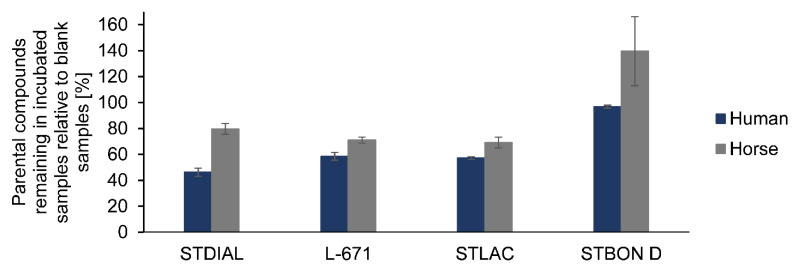
Comparison of parental compounds of the investigated PSDs remaining in the pI metabolism approaches containing human respective horse microsomes after 90 min incubation of 0.04 mg/mL PSD. Values were determined in duplicate by HPLC-Orbitrap-HRMS and are displayed relative to the “blank sample degradation”.

Regarding the pI metabolism of PSDs using horse microsomes, it was observed that the same reactions took place. Therefore, none of the formed metabolites were specific for one species. However, the extent to which metabolites were formed was lower in equine microsomes and not all metabolites formed in the human microsomes were detectable. The transformation of STDIAL and STLAC by horse microsomes was still comparable to human microsomes. In both cases, all but the desaturated metabolites were formed, as it was observed during experiments with human microsomes. L-671 [+O_2_–H_2_] could not be detected, and concerning STBON D, only the metabolites STBON D [+O–H_2_] and STBON D [+O] were identified (compare Table S6 in the Supplementary Materials). In addition, multiple occurrences of signals of the same metabolite were less frequent, and differences between the metabolite spectra of the two species were observable. A conceivable reason for this could be a higher conversion rate of PSDs by human microsomes, which is indicated by a lower number of parental compounds remaining in the respective incubated approaches as shown graphically in Figure 3. Well-known species-specific differences in terms of content and activity of metabolizing enzymes in the microsomes are also a possible explanation.

#### 2.1.2. Phase II Metabolism of PSDs in Human and Horse Liver Microsomes and Cytosol

**Figure 4:**
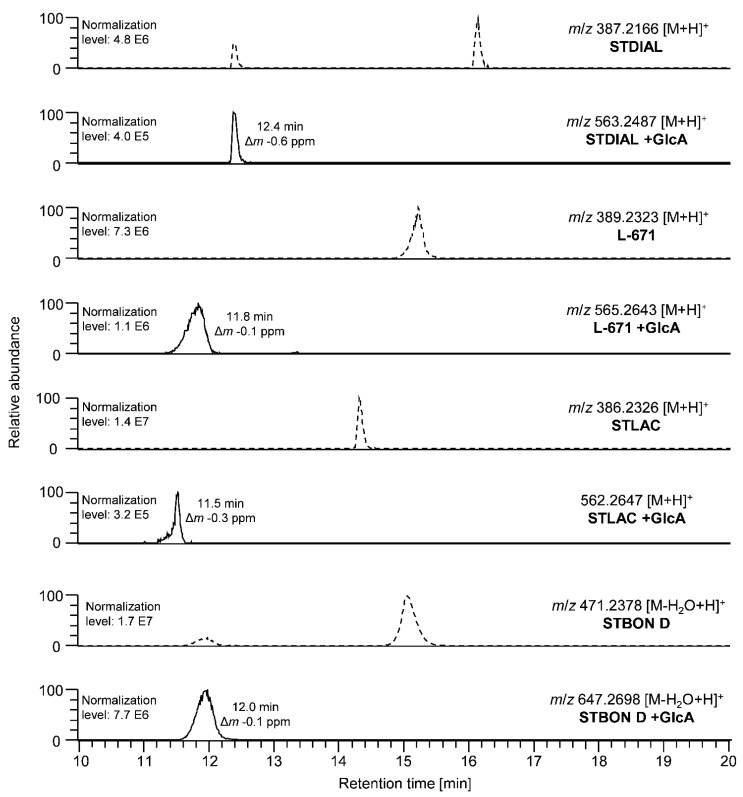
Excerpts of HPLC-Orbitrap-HRMS chromatograms of measurements of the hepatic pII metabolism experiments (glucuronidation) with human microsomes incubated with STDIAL, L-671, STLAC, and STBON D. XICs (acquired with a mass tolerance of 5 ppm) of parental compounds (dashed lines) followed by XICs of respective glucuronides are displayed. Mass differences (Δ*m*) between theoretical and accurate *m*/*z* are given in ppm.

To study the pII metabolism, PSDs were incubated with liver microsomes and cytosol. The identification of pII metabolites was performed, as described for pI metabolism, based on a mass list containing potential metabolites (Supplementary Materials Table S5). The HPLC-HRMS analysis showed that sulfates and glucuronides for all four investigated PSDs were formed in the human and the equine models. Chromatographic information on the identified glucuronides of the investigated PSDs after incubation with human microsomes is provided in Figure 4. The conversion varied depending on the PSD but, overall, the largest shares of the parental compounds were still detectable in all approaches compared to the control samples “stability”.

In the following, the identification of glucuronidated metabolites is exemplarily described for STBON D. The XICs of STBON D and STBON D +GlcA are shown in Figure 4. The accurate mass of the signal at 12.0 min matched the exact mass of STBON D +GlcA (*m*/*z* 647.2698) with a low mass deviation of Δ*m* −0.1 ppm. An in-source fragmentation was an additional confirmation of the glucuronidated metabolite, as it partially provoked a cleavage of the glucuronic acid moiety, resulting in a neutral loss of C_6_H_8_O_6_ (see Figure S4, Supplementary Materials). Furthermore, this led to a second signal in the XIC of the parental compound in Figure 4 at a retention time of the respective glucuronide. The determination of the exact binding position of the glucuronic acid at the STBON D backbone was not possible based on the MS data but was achieved by nuclear magnetic resonance spectroscopy (NMR) analysis of the isolated STBON D +GlcA as described below. The complete list of identified glucuronides including retention times and mass errors is presented in Table S7 of the Supplementary Materials for both species. The conversion of PSDs during the pII metabolism studies by microsomes/cytosol of the two species is discussed below.

**Figure 5:**
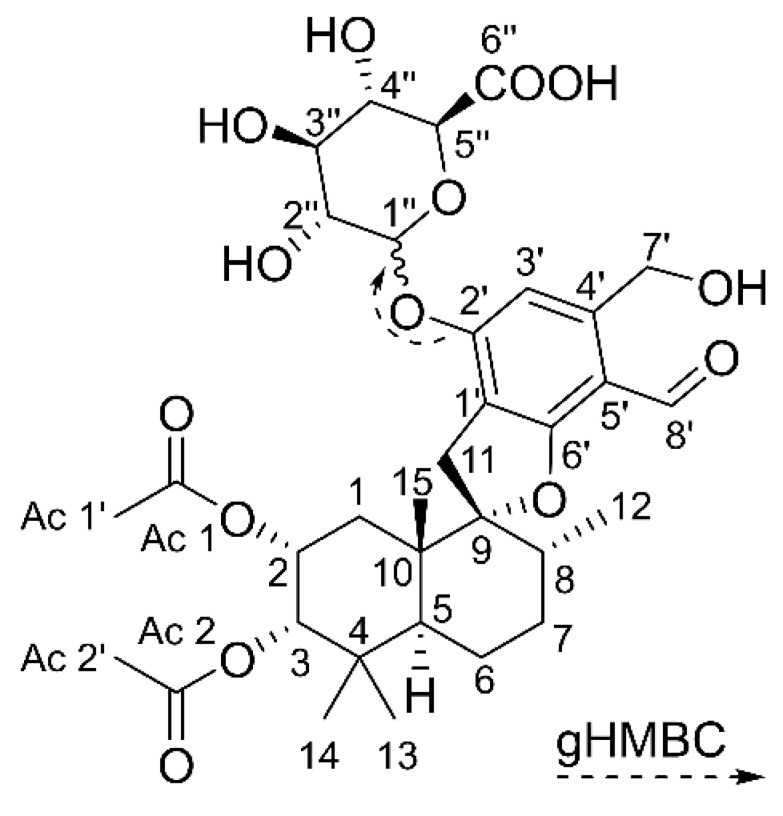
Determined chemical structure of pII metabolite STBON D +GlcA isolated from horse liver microsomes.

In order to identify the exact binding position of the glucuronic acid, STBON D +GlcA was isolated from a large-scale approach and the structure was fully characterized by NMR. For the preparative glucuronidation of STBON D, self-prepared horse liver microsomes were used to reduce the amount of used commercial human biological material. The parameters of the incubation conditions were optimized and upscaled to increase the yield of STBON D +GlcA (compare Table S8, Supplementary Materials). Finally, STBON D +GlcA was prepurified by reversed-phase solid-phase extraction and isolated using reversed-phase HPLC with ultraviolet detection at 225 nm. The structure elucidation was achieved by one- and two-dimensional NMR measurements. Based on the coupling in the gHMBC NMR spectrum between the C-2′ carbon (δ 156.8 ppm) and the proton, which is bound to the anomeric C-1″ carbon of the glucuronic acid (δ 4.96 ppm), the binding position of glucuronic acid was unequivocally determined to be at C-2′. The glucuronic acid conformation at C-1″ (α or β) could not be characterized on the basis of coupling constants as described by Schmidt et al. [31], as the proton signals of H-1″ and H-2″ showed multiplets. The NMR data are summarized in Table 1. The corresponding elucidated structure of STBON D +GlcA is displayed in Figure 5.

**Figure 6:**
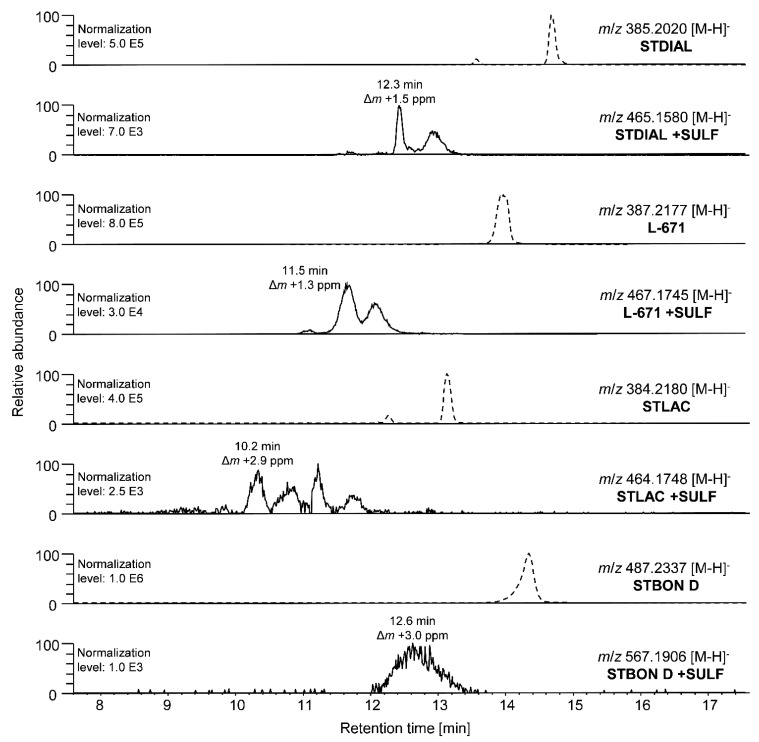
Excerpts of HPLC-QTOF-HRMS chromatograms of measurements of the hepatic pII metabolism experiments (sulfation) with human microsomes incubated with STDIAL, L-671, STLAC, and STBON D. XICs (acquired with a mass tolerance of 0.005 Da) of parental compounds (dashed lines) followed by XICs of respective sulfates are displayed. Mass differences (Δ*m*) between theoretical and accurate *m*/*z* are given in ppm.

In the course of hepatic pII metabolism, a formation of PSD-sulfates was not observable by HPLC-Orbitrap-HRMS measurements. Therefore, samples from sulfation experiments were also applied to a QTOF-HRMS instrument, which enabled the detection of potential sulfates with higher sensitivity. The corresponding HPLC-QTOF chromatograms after incubation of all investigated PSDs with human cytosol are shown in Figure 6. The most abundant sulfate signals were observed after the conversion of L-671. Here, two signals in the XIC of L-671 +SULF can be observed, indicating a sulfation of L-671 at different positions of the molecule and the formation of chromatographically separable isomers. Comparable observations were made for the metabolites STDIAL +SULF and STLAC +SULF. The identification of sulfated metabolites was carried out in analogy to the identification of PSD-glucuronides. The more abundant signal of L-671 +SULF eluted at a retention time of 11.5 min and the accurate mass showed little deviation to the exact mass of L-671 +SULF. Additionally, in-source fragmentation partially provoked a neutral loss of SO_3_. The associated MS^1^ spectrum is presented in Figure S5 in the Supplementary Materials. The determination of the exact binding positions of the sulfate moiety at the PSD backbone was not possible based on the recorded MS data. The complete list of identified sulfates, including retention times and mass errors, is presented in Table S9, Supplementary Materials.

**Figure 7:**
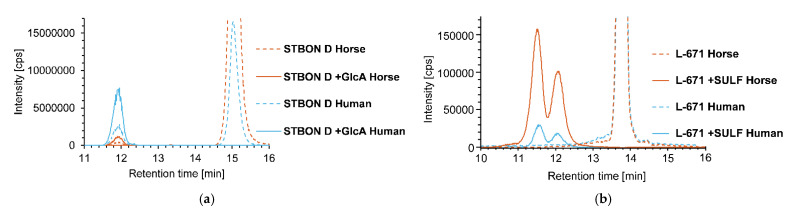
Conversion of PSDs during in vitro pII metabolism experiments applying human and horse microsomes/cytosol: (**a**) HPLC-Orbitrap-HRMS chromatograms of the hepatic pII metabolism experiments (glucuronidation) with human and horse microsomes incubated for 90 min with STBON D. XICs (acquired with a mass tolerance of 5 ppm) of the [M-H_2_O+H]^+^ adduct of STBON D (*m*/*z* 471.2378) and STBON D-glucuronide (STBON D +GlcA) (*m*/*z* 647.2698) are displayed. (**b**) HPLC-QTOF-HRMS chromatograms of hepatic pII metabolism experiments (sulfation) with human and horse microsomes incubated with L-671. XICs (acquired with a mass tolerance of 0.005 Da) of the [M-H]^−^ of L-671 (*m*/*z* 387.2177) and L-671 +sulfate (L-671 +SULF) (*m*/*z* 467.1745) are displayed.

Comparing the pII metabolism of the two investigated species, differences concerning glucuronidation and sulfation of PSDs were observed. During glucuronidation experiments, human microsomes showed higher conversion rates, whereas an increased formation of sulfates was observed with horse cytosol, indicating species-specific differences, again. These findings are presented exemplarily for the PSDs STBON D (glucuronidation) and L-671 (sulfation) in Figure 7. Figure 7a shows higher signals for STBON D +GlcA and lower signals of the respective parental compound using human microsomes compared to horse microsomes. Opposite observations were made for the conversion to L-671 +SULF in Figure 7b.

## 3. Conclusions

Fungal species of the genus *Stachybotrys* have been reported to be involved in cases of (pulmonary) illness and disease after exposure in indoor environments and are, therefore, suspected to exhibit toxic potential. The role of the different mycotoxins in clinical symptoms of stachybotryotoxicosis, especially the one of the abundant compound class of PSDs, is still not sufficiently described. For this reason, the major objective of the presented studies was to gain insight into the potential metabolic conversion of PSDs.

Studies on hepatic metabolism in vitro revealed the formation of pI and pII metabolites. The results depended both on the chemical structure of the applied PSD and the species of the hepatic biological material. Overall, higher potential for metabolic conversion of PSDs was determined for human liver compared to equine liver fractions. The largest variation in potential pI metabolites was detected for L-671 in HPLC-Orbitrap-HRMS experiments. Hydroxylation and oxidation reactions were detected for all investigated PSDs. Due to the low yield of the observed pI metabolites, additional structure elucidation by NMR after isolation was not possible. Concerning pII metabolism, the formation of sulfates and glucuronides was confirmed for all investigated PSDs. In the case of glucuronides of the four PSDs, only one intense signal was observed in HPLC-Orbitrap-HRMS detection per PSD. The binding position of the glucuronic acid moiety was determined exemplarily for STBON D +GlcA at the phenolic 2′-OH of the PSD backbone using NMR after upscaling and purification of the product. As multiple peaks were observed after HPLC-QTOF-HRMS analysis of sulfated metabolites of the four PSDs, a specific binding site of the sulfate moiety could not be identified.

Currently, the mode of action of this class of *Stachybotrys* mycotoxins is unknown and remains an interesting subject for further studies in order to characterize the toxicity as well as potential structure–bioactivity relationships of PSDs with different substitution patterns at the basic backbone. Regarding airborne exposure to *Stachybotrys* and associated respiratory uptake of PSDs, further cytotoxicity testing could be carried out using cells from the respiratory tract to investigate the influences of a respiratory exposure route. However, as most cells from the respiratory tract are incapable of xenobiotic metabolism, further experiments with metabolically competent cells, such as hepatocarcinoma cells or primary human hepatocytes, should be carried out taking the effect of pI and pII metabolites of PSDs into account [32,33]. Moreover, combinatory effects of different PSDs respective of other compounds produced by *Stachybotrys* have not been studied so far but should be included in future research, as these compounds frequently co-occur.

## 4. Materials and Methods

### 4.1. Materials, Chemicals, and Reagents

Solvents and acids were purchased from Fisher Scientific GmbH (Schwerte, Germany), Grüssing (Filsum, Germany), Carl Roth GmbH & Co. KG (Karlsruhe, Germany), Merck KGaA (Darmstadt, Germany), VWR (Darmstadt, Germany), and ARMAR Chemicals (Döttingen, Switzerland). Water was purified by an ELGA PURELAB system (Veolia Water Technologies, Celle, Germany). Other chemicals were obtained from Carl Roth GmbH & Co. KG (Karlsruhe, Germany), Sigma-Aldrich GmbH (Steinheim, Germany), VWR International GmbH (Darmstadt, Germany), Merck KGaA (Darmstadt, Germany), and Fluka^®^ Analytical (Seelze, Germany). All mycotoxin standard substances were isolated and analytically characterized according to Jagels et al. [34]. Fresh horse liver was purchased locally and frozen in liquid nitrogen. Until further usage, the liver was stored at −80 °C. The preparation of microsomes and cytosol was carried out according to Lake et al. [35] and the protein content was determined by Bradford assay with bovine serum albumin as a reference [36]. Human liver microsomes and cytosol (Ultrapool^TM^) were provided by Corning, Inc. (Corning, New York, NY, USA).

### 4.2. Incubation with Liver Microsomes and Cytosol

#### 4.2.1. Incubation with NADPH-Regenerating System

The reaction mixture contained nicotinamide adenine dinucleotide phosphate (NADP^+^) (0.5 mg/mL), glucose-6-phosphate (G6P) (10 mM), G6P dehydrogenase (G6P-DH) (2 U/mL), magnesium chloride (MgCl_2_) (0.4 mM), human or horse microsomes (5 mg/mL), and one of the investigated PSDs (0.04 mg/mL) in addition to NaH_2_PO_4_ buffer (pH 7.4, 85.5 mM). Before incubation, the toxin solutions were transferred to a reaction vessel and the solvent was evaporated. The NADPH-regenerating system (consisting of NADP^+^, G6P and G6P-DH) was prepared in a separate vessel and stored on ice until use, which also applied to the microsomes. Given concentrations represent final values in the reaction mixture. The toxins were resuspended in buffer and MgCl_2_, the NADPH-regenerating system and, finally, the microsomes were added resulting in a total volume of 200 µL per approach. Incubation was carried out on a laboratory shaker (300 rpm) at 37 °C for 90 min. To quench the reaction and to precipitate proteins, 400 µL of acetonitrile (MeCN) (−20 °C) were added. Afterwards, the reaction mixtures underwent centrifugation (15,000× *g*, 4 °C, 15 min) and supernatants were transferred to 1.5 mL HPLC glass vials and directly applied to HPLC-Orbitrap-HRMS analysis as described below. During the pI experiments, harmane served as the positive control. The system was classified as functional when hydroxylated harmane metabolites were formed [37]. Influences of the matrix and the NADPH-regenerating system as well as stability of the PSDs during incubation were considered with three separate control samples (Table S1, Supplementary Materials). All experiments were performed in duplicate.

#### 4.2.2. Incubation with UDPGA and PAPS

Similar approaches were pursued to elucidate the pII metabolism of PSDs. For both glucuronidation and sulfation, the solvent of the PSD stock solutions was evaporated in compliance with the earlier mentioned procedure during the pI metabolism studies. In glucuronidation approaches, NaH_2_PO_4_ buffer (85.5 mM), uridine diphosphate glucuronic acid (UDPGA, 0.3 mM), and MgCl_2_ (0.4 mM) were added to the mycotoxin, followed by human or horse microsomes. The reaction mixtures contained 0.02 mg/mL PSD and had a final volume of 200 µL.

Mycotoxins in sulfation experiments were resuspended in a NaH_2_PO_4_ buffer with a higher concentration (285.0 mM). The mixture was combined with a dithiothreitol (DTT) (1 mM) solution containing ascorbic acid (0.05 mg/mL) and a 3′-phosphoadenosine-5′-phosphosulfate (PAPS) (0.1 mM) solution, followed by cytosol of the species horse and human (each 2.0 mg/mL), resulting in a volume of 100 µL for every reaction approach.

Incubation parameters were chosen as described above. After incubation, the reactions were terminated with MeCN (−20 °C, 400 µL for glucuronidation, 200 µL for sulfation), samples were centrifuged at 15,000× *g* and 4 °C for 15 min. Supernatants were transferred and analyzed by HPLC-Orbitrap-HRMS. Supernatants of sulfation experiments were additionally examined by HPLC-QTOF-HRMS.

Functionality of the glucuronidation and sulfation system were confirmed using 7-hydroxycoumarin as a positive control. The systems were classified as functional when the formation of 7-hydroxycoumarin glucuronide, respectively, 7-hydroxycoumarin sulfate, was observed [38]. Influences of the matrix components and the stability of PSDs during incubation were investigated with two separate control samples (Tables S2 and S3, Supplementary Materials).

### 4.3. Isolation of STBON D +GlcA and Acquisition of Structural Data

For preparative isolation of glucuronidated STBON D (STBON D +GlcA), the standardized glucuronidation approach was optimized to achieve higher yields. STBON D concentration and incubation time were varied and MeCN was added as a solubilizer. Furthermore, it was tested whether an upscaling for preparative application was possible. Concentrations of cofactors and buffers were kept comparable to the ones described above. Horse microsomes were used for metabolization due to their availability. The detailed composition of the preparative approach is shown in Table S8 in the Supplementary Materials.

Prior to incubation, MgCl_2_ (0.4 mM), UDPGA (0.3 mM), and MeCN (4.6%) were mixed into 85.5 mM NaH_2_PO_4_ buffer (pH 7.4). STBON D (0.02 mg/mL) and microsomes (1.05 mg/mL), which were thawed on ice beforehand, were added resulting in a total incubation volume of 262.88 mL, divided into 53 single reaction mixtures of 4.96 mL, each. After mixing thoroughly, the latter were incubated for 24 h at 37 °C on a laboratory shaker (300 rpm). The reaction was quenched by adding 5 mL MeCN (−20 °C) per reaction mixture. Subsequent to a centrifugation step (857× *g*, 4 °C, 15 min), supernatants were combined and prepurified using a solid-phase extraction cartridge (Oasis^®®^ HLB 12 cc, Waters Corporation, Milford, OH, USA). Fractionated elution was performed applying mixtures of MeCN/H_2_O (0–100% MeCN). Aliquots of each fraction were analyzed by HPLC-Orbitrap-HRMS and fractions containing STBOND +GlcA were combined. Afterwards, the majority of the solvent was removed using a rotary evaporator at 37 °C. Isolation and purification of the glucuronide was achieved by semipreparative HPLC-UV (LC-NetII/ADC Jasco Labor und Datentechnik, Gross-Umstadt, Germany). For separation an Eclipse XDB-C18 column (250 × 9.4 mm, 5 µm; Agilent, Ammerbuch, Germany) and a linear gradient (flow rate: 5 mL/min) consisting of MeCN/H_2_O starting at 10% MeCN and finishing at 50% MeCN at 30 min were applied. Detection was performed at a wavelength of 225 nm. A total amount of 1.34 mg STBON D +GlcA was obtained as a slightly yellowish residue with a purity of 90% (based on HPLC diode array detection (DAD)). This equals a relative yield of 18.7%. Accurate mass and fragmentation patterns were determined with HPLC-Orbitrap-HRMS analysis, and structural elucidation was performed by NMR (600 MHz) (Agilent Technologies, Ratingen, Germany). MestReNova 9.0 (Mestrelab Research S. L., Santiago de Compostela, Spain) was used for data analysis.

### 4.4. Instruments, Chromatographic, and Mass Spectrometric Conditions

#### 4.4.1. HPLC-Orbitrap-HRMS

The HPLC-Orbitrap-HRMS analysis of samples was performed by a Nexera XR system (Shimadzu Duisburg, Germany) coupled to a SPD-M30A PDA (Shimadzu Duisburg, Germany) and a LTQ Orbitrap XL (Thermo Fisher Scientific, Walthma, MA, USA) with heated electrospray ionization (HESI) operated in the positive or negative ionization mode.

Chromatographic separation was carried out using a ReproSil-Gold C18 column (150 × 2 mm, 3 µm particle size, Dr. A. Maisch HPLC GmbH, Ammerbuch, Germany) in combination with a 4 × 2 mm universal RP guard column (Macherey-Nagel, Düren, Germany) at a column oven temperature of 40 °C. A volume of 10 µL was injected and a binary gradient of MeCN (A) and water (B) (each +0.1% FA) was applied at a constant flow rate of 0.3 mL/min. The utilized gradient was as follows: 0.0 min, 10% A; 2.0 min, 10% A; 20.0 min, 100% A; 25.0 min, 100% A; 25.1 min, 10% A; 30.0 min, 10% A. To ensure an improved peak-shape of STBON D +GlcA, as presented in Figure 4 and Figure 7a, chromatographic separation was performed using an Ascentis RP-Amide column (150 × 2.1 mm, 5 µm particle size, Supelco Analytical, Darmstadt, Germany) in these particular cases. All other parameters remained unchanged.

The vaporizer temperature in the source was set to 350 °C, and the capillary temperature in the mass spectrometer was adjusted to 275 °C. Voltages of source, capillary, and tube lens were set to 3.5 kV, 20 V, and 125 V in positive ionization mode. Values in the negative mode acquisition were set to −3.5 kV, −35 V, and −110 V, respectively. Full-scan data were recorded at a resolution of 30,000 at *m*/*z* 200–800. MS^2^ data were recorded in the data-dependent acquisition mode using higher-energy collisional dissociation and collision-induced dissociation. Known fragmentation patterns of PSD (basic) structures were used for additional confirmation of metabolites. The software Xcalibur^TM^ (Thermo Fisher Scientific, Dreieich, Germany) was used for the operation of the mass spectrometer, data acquisition, and partly for data processing (Qual Browser). In addition, data processing was executed with the software Skyline (MacCross Lab Software, Seattle, WA, USA).

#### 4.4.2. HPLC-QTOF-HRMS

The analysis of the samples of the sulfation experiments was realized by an Elute HPG 1300 HPLC system in combination with an impact II QTOF mass spectrometer (Bruker Daltonics, Bremen, Germany).

The column, solvents, column oven temperature, and gradient used for chromatographic separation matched the ones described in the HPLC-Orbitrap-HRMS section. The injection volume was increased to 40 µL.

Ionization in the mass spectrometer was performed in an Apollo II ion source (Bruker Daltonics, Bremen, Germany), which was operated separately in the positive and negative electrospray ionization (ESI) mode set at 4.5 and −3.0 kV. Dry gas temperature was set to 220 °C. A mass range of *m*/*z* 100–1000 was covered and full-scan and MS^2^ data were recorded at a spectra rate of 6 Hz. Data-dependent acquisition in the auto MS/MS mode was chosen for the MS^2^ experiments, with a number of three precursors and a signal threshold of 48 counts/1000 cts. Fragmentation took place in a collision-induced dissociation cell. Isolation width and fragmentation energy were automatically adapted to the respective *m*/*z* of the precursor by the software. Sodium formiate solution was used for instrument mass calibration and for recalibration of each data file by implementing a calibration segment at the beginning of each run. The software, Compass HyStar and Compass otofControl (software versions 4.1, Bruker Daltonics, Bremen, Germany), were used for the operation of the HPLC system, the mass spectrometer, and for data acquisition. Data processing was executed with the software DataAnalysis 4.4 and TASQ 2.1 (Bruker Daltonics, Bremen, Germany).

## Figures and Tables

**Table 1:** NMR data of isolated STBON D +GlcA (CD_3_CN; 600 MHz).

**Position**	**δ_C_ (ppm),** **Type**	**δ_H_ (ppm),** **Type (J in Hz)**	**Position**	**δ_C_ (ppm),** **Type**	**δ_H_ (ppm),** **Type (J in Hz)**
1 ^1^	31.5, CH_2_	1.43, 1.52, m	1′	116.5, C	-
2	69.7, CH	5.23, m	2′	156.8, C	-
3	78.4, CH	4.92, m	3′	100.3, CH	6.57, s
4	39.2, C	-	4′	147.6, C	-
5	42.0, CH	2.17, m	5′	112.9, C	-
6 ^1^	21.5, CH_2_	1.52, 1.60, m	6′	157.7, C	-
7 ^1^	32.1, CH_2_	1.52, 1.60, m	7′	63.8, CH_2_	4.87, m
8	37.7, CH	1.94, m	8′	189.7, CH	10.35, s
9	100.9, C	-	1′′	102.1, CH	4.96, m
10	45.0, C	-	2′′ ^2^	74.7, CH	3.50, m
11	33.1, CH_2_	2.94, 3.34, m	3′′ ^2^	77.8, CH	3.50, m
12	15.8, CH_3_	0.81, d (6.5)	4′′	73.6, CH	3.53, m
13	22.0, CH_3_	1.04, s	5′′	76.6, CH	3.76, m
14	28.4, CH_3_	0.93, s	6′′	n.d. ^3^, C	-
15	17.2, CH_3_	1.16, s			
Ac 1	172.3, C	-			
Ac 1′	21.0, CH_3_	1.86, s			
Ac 2	172.4, C	-			
Ac 2′	20.8, CH_3_	2.00, s			

^1^ Signals of protons at positions 1, 6, and 7 are overlapping. ^2^ Signals of protons at positions 2″ and 3″ are overlapping. ^3^ n.d.: Not detectable.

## Data Availability

Data are presented in the manuscript and in the Supplementary Materials. Additional data are available upon request from the corresponding author (humpf@uni-muenster.de).

## Supplementary Materials

The following are available online at https://www.mdpi.com/article/10.3390/toxins14060395/s1, Table S1: Composition of the control samples of the pI metabolism experiments; Table S2: Composition of the control samples of the pII metabolism experiments (glucuronidation); Table S3: Composition of the control samples of the pII metabolism experiments (sulfation); Table S4: Sum formulas of the investigated PSDs; Table S5: Investigated possible metabolic reactions in the course of the pI and pII metabolisms and applied nomenclature of identified possible metabolites; Table S6: Identified possible metabolites formed in the course of the hepatic pI experiments; Table S7: Identified glucuronidated PSD metabolites formed in the case of the hepatic pII experiments; Table S8: Composition of the preparative approach for the preparation of STBON D +GlcA; Table S9: Identified sulfated PSD metabolites formed in the course of hepatic pII experiments; Figure S1: HPLC-DAD-Orbitrap-HRMS chromatograms of measurement of hepatic pI metabolism experiments with human microsomes incubated with STDIAL; Figure S2: HPLC-DAD-Orbitrap-HRMS chromatograms of measurement of the hepatic pI metabolism experiments with human microsomes incubated with STLAC; Figure S3: HPLC-DAD-Orbitrap-HRMS chromatograms of measurement of the hepatic pI metabolism experiments with human microsomes incubated with STBON D; Figure S4: MS^1^ spectrum of STBON D +GlcA; Figure S5: MS^1^ spectrum of L-671 +SULF.
