# Supplementary material for: In Vitro Metabolism of Phenylspirodrimanes Derived from the Indoor Fungus Stachybotrys
Source: Toxins (Basel). 2022 Jun 8;14(6):395. doi: 10.3390/toxins14060395 (PMC9227918; doi:10.3390/toxins14060395)
Supplement: Supplementary file 1 [file toxins-14-00395-s001.zip › toxins-1744192-supplementary.pdf]

In Vitro Metabolism of Phenylspirodrimanes Derived from the Indoor Fungus *Stachybotrys*

Viktoria Lindemann, Annika Jagels †, Matthias Behrens, Florian Hübner and Hans-Ulrich Humpf *

Table S1. Composition of control samples of pI metabolism experiments.

Substance	Control sample		
	Matrix	Stability	Degradation
NADP	✓	✓	✗
G6P	✓	✓	✗
G6P-DH	✓	✓	✗
$MgCl_2$	✓	✓	✓
Buffer (NaH_2PO_4)	✓	✓	✓
Mycotoxin	✗	✓	✓
Microsomes	✓	✗	✓

Table S2. Composition of control samples of pII metabolism experiments (glucuronidation).

Substance	Control sample	
	Matrix	Stability
UDPGA	✓	✓
$MgCl_2$	✓	✓
Buffer (NaH_2PO_4)	✓	✓
Mycotoxin	✗	✓
Microsomes	✓	✗

Table S3. Composition of control samples of pII metabolism experiments (sulfation).

Substance	Control sample	
	Matrix	Matrix
DTT	✓	✓
PAPS	✓	✓
Buffer (NaH_2PO_4)	✓	✓
Mycotoxin	✗	✓
Cytosol	✓	✗

Table S4. Sum formulas of investigated PSDs.

Analyte	Sum formula
L-671	$C_{23}H_{32}O_5$
STBON D	$C_{27}H_{36}O_8$
STDIAL	$C_{23}H_{30}O_5$
STLAC	$C_{23}H_{31}NO_4$

Table S5. List of investigated possible metabolic reactions in the cause of pI and pII metabolism, correlating changes in sum formula of the respective PSDs and applied nomenclature of identified possible metabolites.

Phase of metabolism	Metabolic reaction	Change in sum formula (general)	Change in sum formula (exact)	Suffix
I	Oxidation, Hydroxylation, Epoxidation	+O	[$+O_2-H_4$]<br>[$+O-H_4$]<br>[$+O_2-H_2$]<br>[+O]	[$+O_2-H_4$]<br>[$+O-H_4$]<br>[$+O_2-H_2$]<br>[+O]
	Deoxidation	-O	[–O]<br>[$-O_2$]<br>[$-O_3$]	n. d.[1]
	Reduction	$+H_2$	[$+H_2$]<br>[$+H_4$]	n. d.
	Desaturation[2]	$-H_2$	[$-H_2$]<br>[$-H_4$]<br>[$-H_6$]	n. d.<br>[$-H_4$]<br>[$-H_6$]
	Demethylation	$-CH_2$	[$-CH_2$]<br>[$-C_2H_4$]<br>[$-C_3H_6$]<br>[$-C_4H_8$]	n. d.
	Deacetylation[3]	$-C_2H_2O$	[$-C_2H_2O$]<br>[$-C_4H_4O_2$]	n. d.
II	Glucuronidation	$+C_6H_8O_6$	[$+C_6H_8O_6$]	+GlcA
	Sulfation	$+SO_3$	[$+SO_3$]	+SULF
	Glutathione conjugation	$+SC_{10}H_{15}N_3O_6$	[$+SC_{10}H_{15}N_3O_6$]	n. d.

[1]: Not detected.

[2]: Primarily detected in combination with oxidative reactions.

[3]: Only analyzed for STBON D.

Table S6. List of identified possible metabolites formed in the cause of hepatic pI experiments including analytical data.[4]

Species	Name	Sum formula (neutral)	Retention time [min]	Theroetical *m/z* [M+H]+	Mass deviation of measured *m/z* [ppm]
Human	STDIAL [$+O_2–H_4$]	$C_{23}H_{26}O_7$	13.2	415.1751	-1.0
	STDIAL [$+O–H_2$]	$C_{23}H_{28}O_6$	12.6	401.1959	-0.8
	STDIAL [$+O_2–H_2$]	$C_{23}H_{28}O_7$	12.1	417.1908	-0.7
	STDIAL [+O]	$C_{23}H_{30}O_6$	11.8	403.2115	-1.5
	L-671 [$+O_2–H_4$]	$C_{23}H_{28}O_7$	11.8	417.1908	+1.9
	L-671 [$+O–H_2$]	$C_{23}H_{30}O_6$	11.4	403.2115	+2.5
	L-671 [$+O_2–H_2$]	$C_{23}H_{30}O_7$	11.2	419.2064	+1.7
	L-671 [+O]	$C_{23}H_{32}O_6$	11.1	405.2272	+1.7
	L-671 [$–H_4$]	$C_{23}H_{28}O_5$	11.4	385.2010	+1.9
	L-671 [$–H_6$]	$C_{23}H_{26}O_5$	13.3	383.1853	+1.4
	ST LAC [$+O_2–H_4$]	$C_{23}H_{27}NO_6$	11.1	414.1911	-2.2
	ST LAC [$+O–H_2$]	$C_{23}H_{29}NO_5$	10.8	400.2118	-2.0
	ST LAC [$+O_2–H_2$]	$C_{23}H_{29}NO_6$	8.9	416.2068	-1.0
	ST LAC [+O]	$C_{23}H_{31}NO_5$	10.5	402.2275	-1.5
	STBON D [$+O_2–H_4$]	$C_{27}H_{32}O_{10}$	9.7	517.2068	+0.8
	STBON D [$+O–H_2$]	$C_{27}H_{34}O_9$	10.6	503.2276	+0.4
	STBON D [$+O_2–H_2$]	$C_{27}H_{34}O_{10}$	9.2	519.2225	+0.2
	STBON D [+O][5]	$C_{27}H_{36}O_9$	11.1	487.2326	-0.4
Horse	STDIAL [$+O_2–H_4$]	$C_{23}H_{26}O_7$	13.2	415.1751	+1.1
	STDIAL [$+O–H_2$]	$C_{23}H_{28}O_6$	12.6	401.1959	+1.1
	STDIAL [$+O_2–H_2$]	$C_{23}H_{28}O_7$	12.1	417.1908	+1.4
	STDIAL [+O]	$C_{23}H_{30}O_6$	11.8	403.2115	+0.5
	L-671 [$+O_2–H_4$]	$C_{23}H_{28}O_7$	11.8	417.1908	+1.6
	L-671 [$+O–H_2$]	$C_{23}H_{30}O_6$	11.4	403.2115	+1.8
	L-671 [$+O_2–H_2$]	$C_{23}H_{30}O_7$	-	419.2064	-
	L-671 [+O]	$C_{23}H_{32}O_6$	11.1	405.2272	+2.0
	L-671 [$–H_4$]	$C_{23}H_{28}O_5$	11.4	385.2010	+1.9
	L-671 [$–H_6$]	$C_{23}H_{26}O_5$	13.3	383.1853	+1.1
	ST LAC [$+O_2–H_4$]	$C_{23}H_{27}NO_6$	11.1	414.1911	+1.7
	ST LAC [$+O–H_2$]	$C_{23}H_{29}NO_5$	10.8	400.2118	+2.0
	ST LAC [$+O_2–H_2$]	$C_{23}H_{29}NO_6$	9.6	416.2068	+1.6
	ST LAC [+O]	$C_{23}H_{31}NO_5$	10.5	402.2275	+1.5
	STBON D [$+O_2–H_4$]	$C_{27}H_{32}O_{10}$	-	517.2068	-
	STBON D [$+O–H_2$]	$C_{27}H_{34}O_9$	9.3	503.2276	-0.1
	STBON D [$+O_2–H_2$]	$C_{27}H_{34}O_{10}$	-	519.2225	-
	STBON D [+O][5]	$C_{27}H_{36}O_9$	11.1	487.2326	-0.3

[4] In case of multiple occurring signals, the analytical data of the most abundant one is given.

[5] Theoretical *m/z* of principial ion ([$M-H_2O+H]^+$) is listed. An assignment of this *m/z* to the $[M+H]^+$ of desaturated STBON D is also possible ([$–H_2$]).

Table S7. List of identified glucuronidated PSD metabolites formed in the cause of hepatic pII experiments including analytical data.[6.]

Species	Name	Sum formula (neutral)	Retention time [min]	Theoretical *m/z* $[M+H]^+$	Mass deviation of measured *m/z* [ppm]
Human	STDIAL +GlcA	$C_{29}H_{38}O_{11}$	12.4	563.2487	-0.6
	L-671 +GlcA	$C_{29}H_{40}O_{11}$	11.8	565.2643	-0.1
	STLAC +GlcA	$C_{29}H_{39}NO_{10}$	11.5	562.2647	-0.3
	STBON D-GlcA[6]	$C_{33}H_{44}O_{14}$	12.0	647.2698	-0.1
Horse	STDIAL +GlcA	$C_{29}H_{38}O_{11}$	12.4	563.2487	-0.9
	L-671 +GlcA	$C_{29}H_{40}O_{11}$	11.8	565.2643	-0.1
	STLAC +GlcA	$C_{29}H_{39}NO_{10}$	11.5	562.2647	-0.3
	STBON D +GlcA[6]	$C_{33}H_{44}O_{14}$	12.0	647.2698	±0.0

[6] Theoretical *m/z* of principial ion ($[M-H_2O+H]^+$) is listed.

Table S8. Composition of the preparative approach for the preparation of STBON D +GlcA.

Substance	Concentration of stock solution	Volume in "buffer with additives" [mL]	Volume in approach [mL]	Concentration in preparative approach
UDPGA	15.97 mmol/L	5.10	-	0.32 mmol/L
$MgCl_2$	12.21 mmol/L	9.50	-	0.46 mmol/L
Buffer (NaH_2PO_4)	85.50 mmol/L	225.40	-	76.35 mmol/L
MeCN	100.00 %	12.40	-	4.91%
"Buffer with additives"	-	-	4.67	-
STBON D	4.96 mg/mL	-	1.06	0.02 mg/mL
Horse microsomes	19.3 mg/mL	-	14.31	1.05 mg/mL
Total volume		262.88		

Table S9. List of identified sulfated PSD metabolites formed in the cause of hepatic pII experiments including analytical data.

Species	Name	Sum formula (neutral)	Retention time [min]	Theroetical *m*/*z* [M-H]$^-$	Mass deviation of measured *m*/*z* [ppm]
Human	STDIAL +SULF	$C_{23}H_{30}O_8S$	12.3	465.1589	+1.5
	L-671 +SULF	$C_{23}H_{32}O_8S$	11.5	467.1745	+1.3
	STLAC +SULF	$C_{23}H_{31}NO_7S$	10.2	464.1748	+2.9
	STBON D +SULF	$C_{27}H_{36}O_{11}S$	12.6	567.1906	+3.0
Horse	STDIAL +SULF	$C_{23}H_{30}O_8S$	12.3	465.1589	+0.6
	L-671 +SULF	$C_{23}H_{32}O_8S$	11.5	467.1745	+1.5
	STLAC +SULF	$C_{23}H_{31}NO_7S$	10.2	464.1748	+1.0
	STBON D +SULF	$C_{27}H_{36}O_{11}S$	12.6	567.1906	-1.6

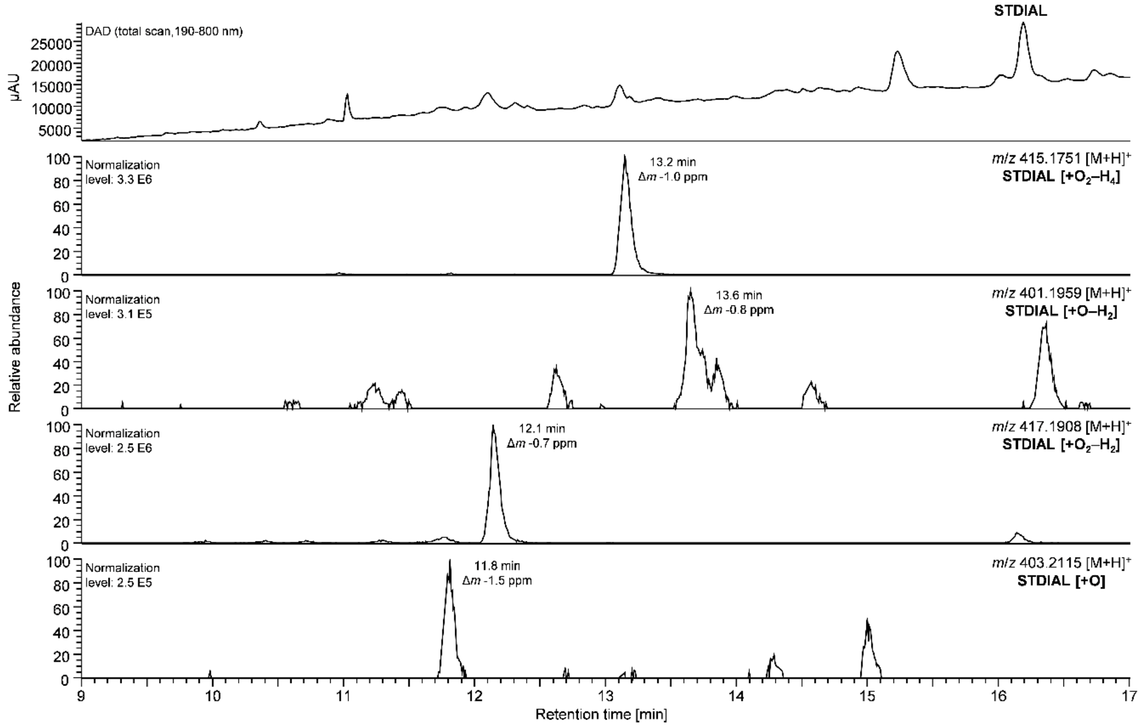

Figure S1. HPLC-DAD-Orbitrap-HRMS chromatograms of the hepatic pI metabolism of STDIAL in human liver microsomes and extracted ion chromatograms (XICs) (acquired with a mass tolerance of 5 ppm) of potential metabolites of STDIAL. In case of multiple occurring signals, the mass deviation (Δ*m*) of the most abundant signal is given.

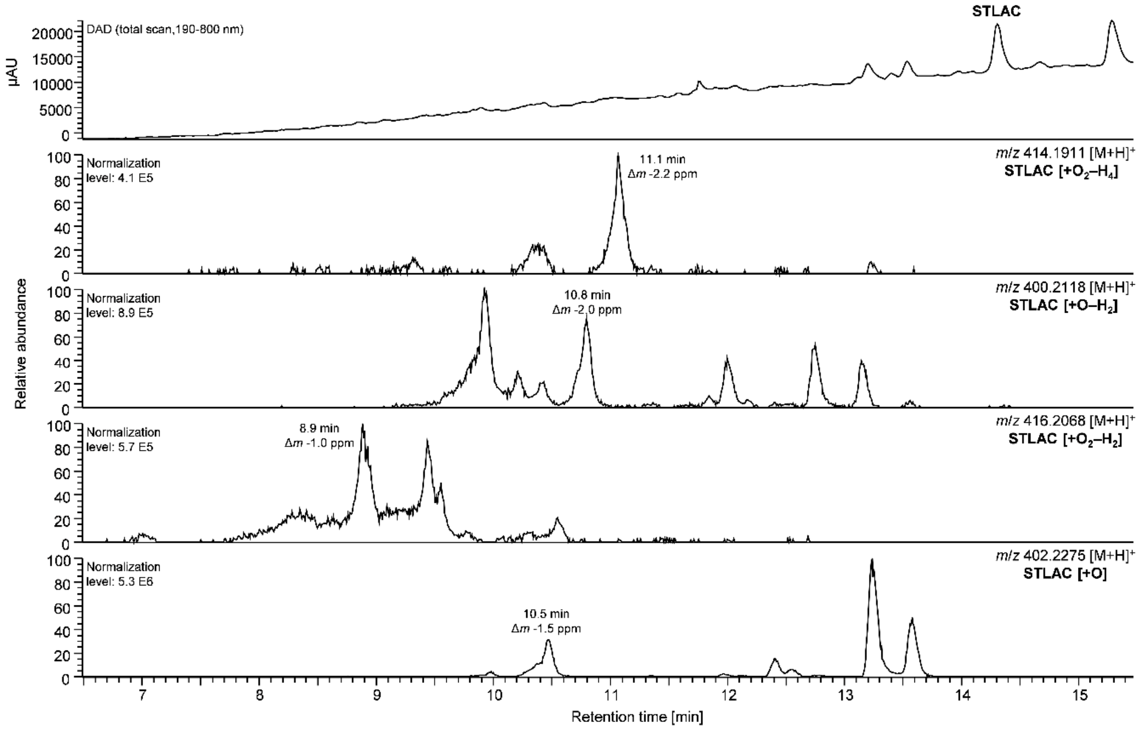

Figure S2. HPLC-DAD-Orbitrap-HRMS chromatograms of the hepatic pI metabolism of STLAC in human liver microsomes and extracted ion chromatograms (XICs) (acquired with a mass tolerance of 5 ppm) of potential metabolites of STLAC. In case of multiple occurring signals, the mass deviation (Δ*m*) of the most abundant signal is given.

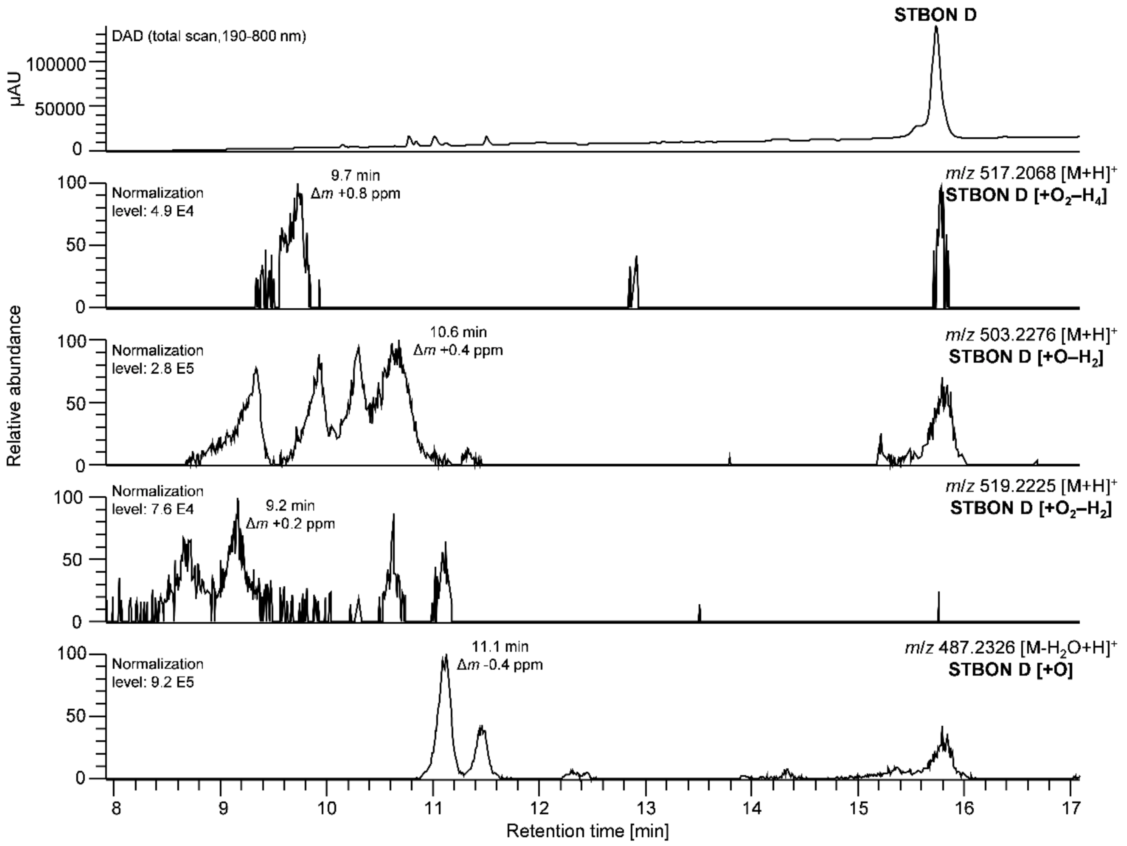

Figure S3. HPLC-DAD-Orbitrap-HRMS chromatograms of the hepatic pI metabolism of STBON D in human liver microsomes and extracted ion chromatograms (XICs) (acquired with a mass tolerance of 5 ppm) of potential metabolites of STBON D. In case of multiple occurring signals, the mass deviation (Δm) of the most abundant signal is given.

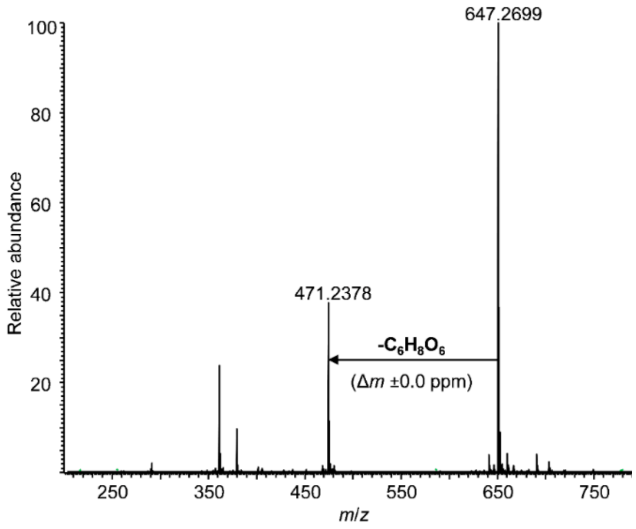

Figure S4. MS[1] spectrum of STBON D +GlcA at a retention time of 12.0 min (compare Figure 4) with labelled in source loss of glucuronic acid (Δm: mass deviation between theoretical and accurate m/z).

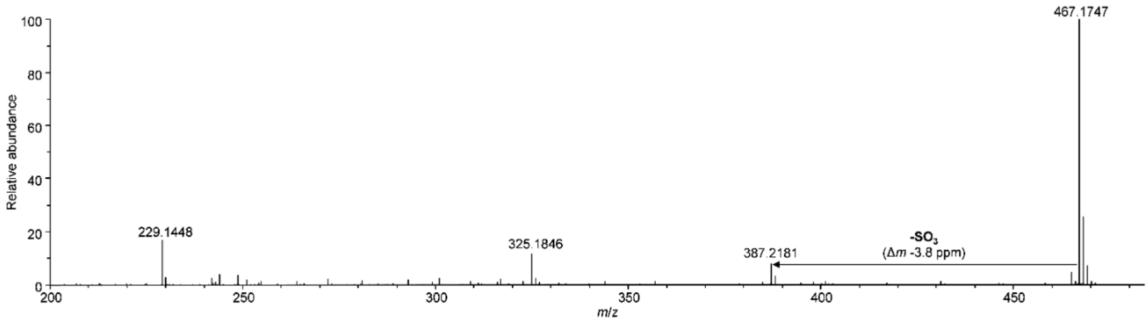

Figure S5: MS[1] spectrum of L-671 +SULF at a retention time of 11.5 min with labelled in source loss of the sulfate moiety. Mass differences (Δ*m*) between theoretical and accurate *m*/*z* are given in ppm.
